# Supplementary material for: Plasticity of source-sink dynamics contributes to wheat yield stability
Source: Nat Commun. 2026 Apr 24;17:3781. doi: 10.1038/s41467-026-72330-x (PMC13109390; doi:10.1038/s41467-026-72330-x)
Supplement: Supplementary file 1 — Supplementary Information [file 41467_2026_72330_MOESM1_ESM.pdf]

Plasticity of source-sink dynamics contributes to wheat yield stability

Tien-Cheng Wang[1], Anna Moritz[2], Mahmoud Mabrouk[1,3], Emilio Villar Alegría[1], Burak Arinalp[1], Eliyeh Ganji[4], Lukas Förter[5], Benjamin Wittkop[5], Eva Herzog[2], Rod J Snowdon[5], Andreas Stahl[4], Tsu-Wei Chen[1*]

[1]Section of Intensive Plant Food Systems, Albrecht Daniel Thaer-Institute of Agricultural and Horticultural Sciences, Humboldt Universität zu Berlin, Germany
[2]Department of Biometry and Population Genetics, Institute of Agronomy and Plant Breeding II, Justus Liebig University, Giessen, Germany
[3]Department of Agronomy, Faculty of Agriculture, Cairo University, Giza, Egypt.
[4]Julius Kuehn Institute (JKI), Federal Research Centre for Cultivated Plants, Institute for Resistance Research and Stress Tolerance, Quedlinburg, Germany
[5]Department of Plant Breeding, IFZ Research Centre for Biosystems, Land Use and Nutrition, Justus Liebig University, Giessen, Germany

*: corresponding authors: tsu-wei.chen@hu-berlin.de

Supplementary Information

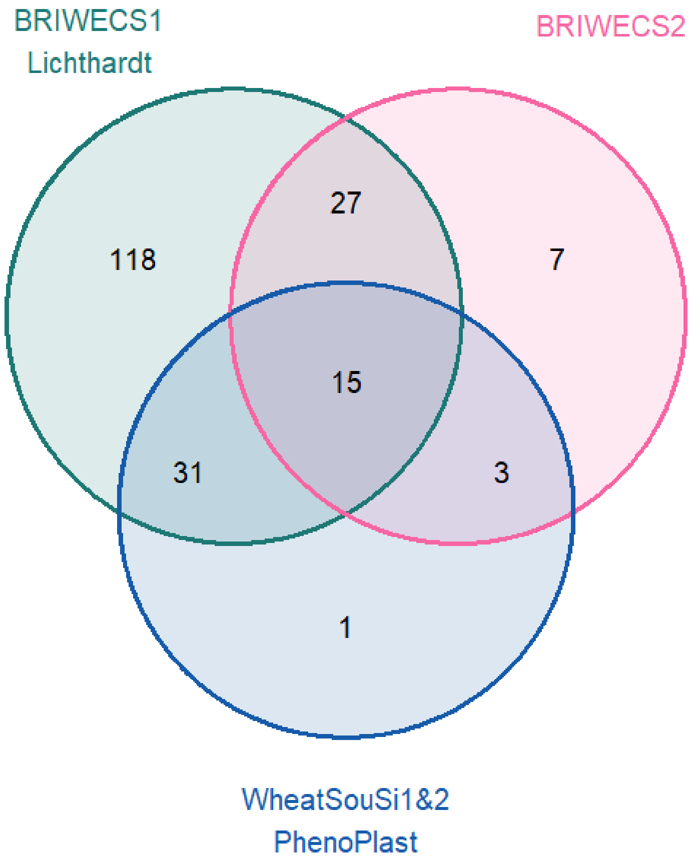

Supplementary Fig. 1. Venn diagram showing the number of cultivars overlaps between different data sources. Details of cultivars are described in Supplementary Table 1.

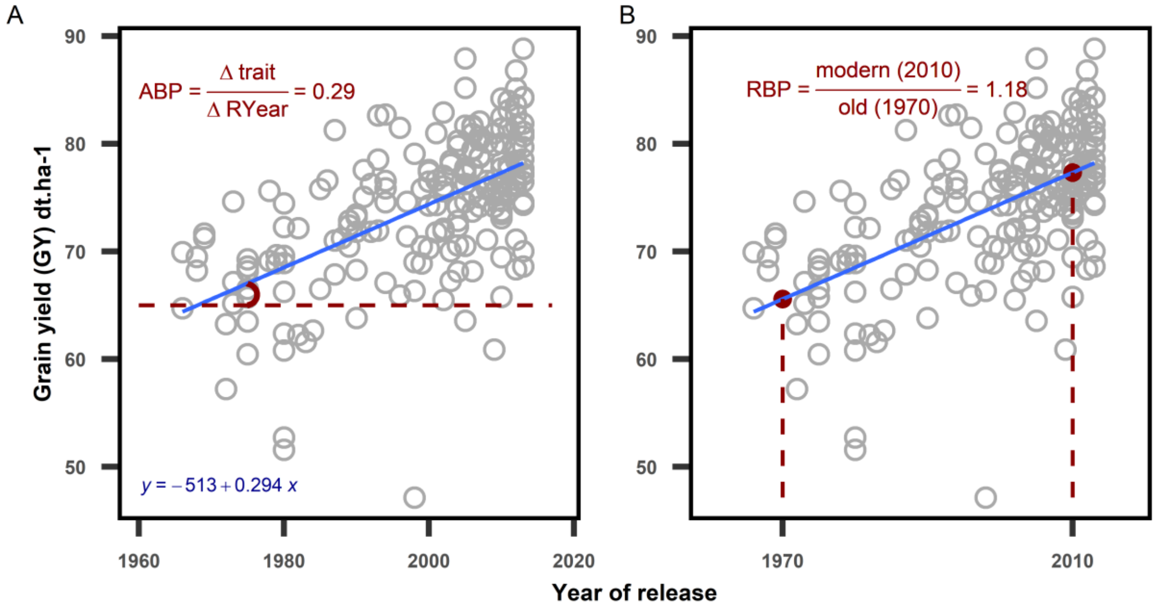

Supplementary Fig. 2. **Examples of the breeding progress in absolute (ABP) and relative (RBP) terms.** A ABP: the slope of regression line (blue) across trait value and year of release (RYear).B RBP: the ratio between estimated performance of modern cultivars released in 2010 and old cultivar released in 1970 based on regression line (blue).

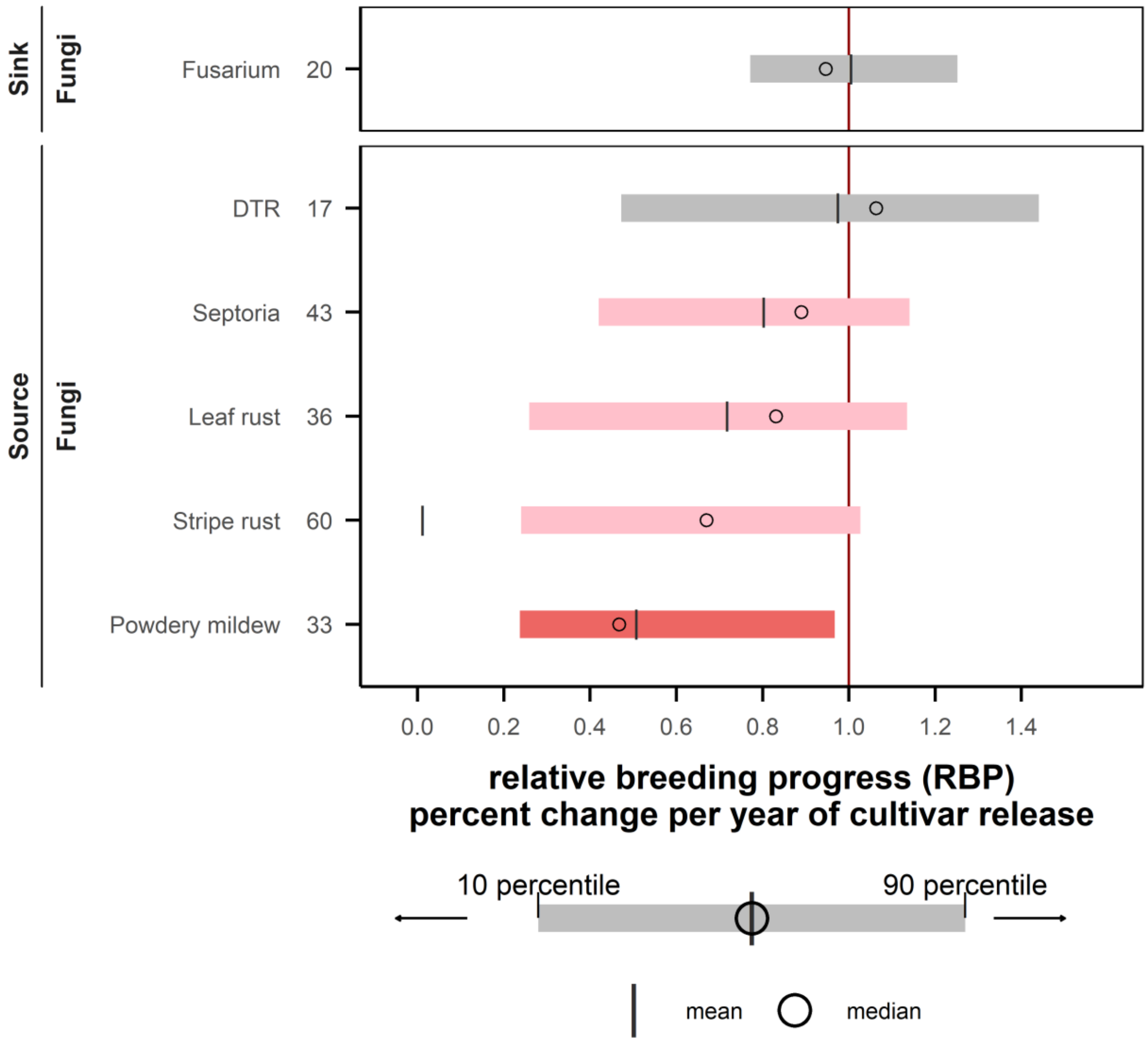

Supplementary Fig. 3. Relative breeding progress (1970–2010) for fungal disease resistance traits. The dark red line ($x = 1$) represents no change over time. Numbers on the *y*-axis indicate the respective number of environments analyzed per trait (combinations of year, location, and treatment across all experiments). Boxplots display the mean (short vertical lines), median (open circle), 10th and 90th percentiles (colored box range), with arrows indicating outliers beyond the *x*-axis range. Red boxes signify a clear decrease in infection, while pink boxes indicate moderate trends. Inconsistent breeding progress for Fusarium and DTR resistance is indicated in gray.

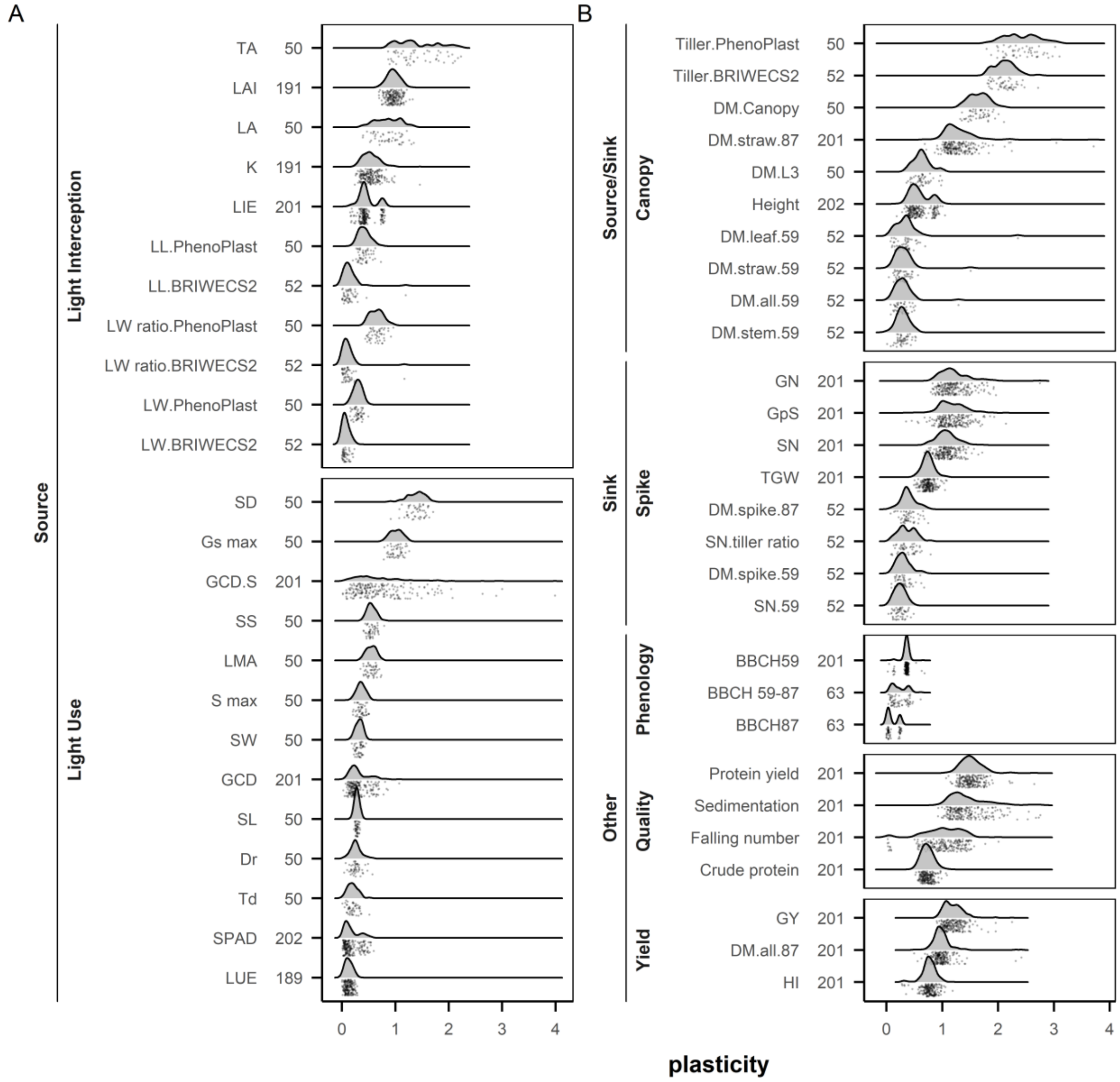

Supplementary Fig. 4. **Plasticity of 51 traits across genotypes, grouped by functional categories.** A source categories. B source/sink, sink and other traits categories. Each trait is displayed with trait abbreviation and number of genotypes. For traits related to leaf morphology (LL: leaf length, LW: leaf width, LW ratio: leaf length to width ratio) and tiller number (Tiller), they are separated for field and control environments to avoid confounding effect of data source on plasticity in trait. Each point represents a genotype's plasticity for a trait; density curves show the distribution of genotypes.

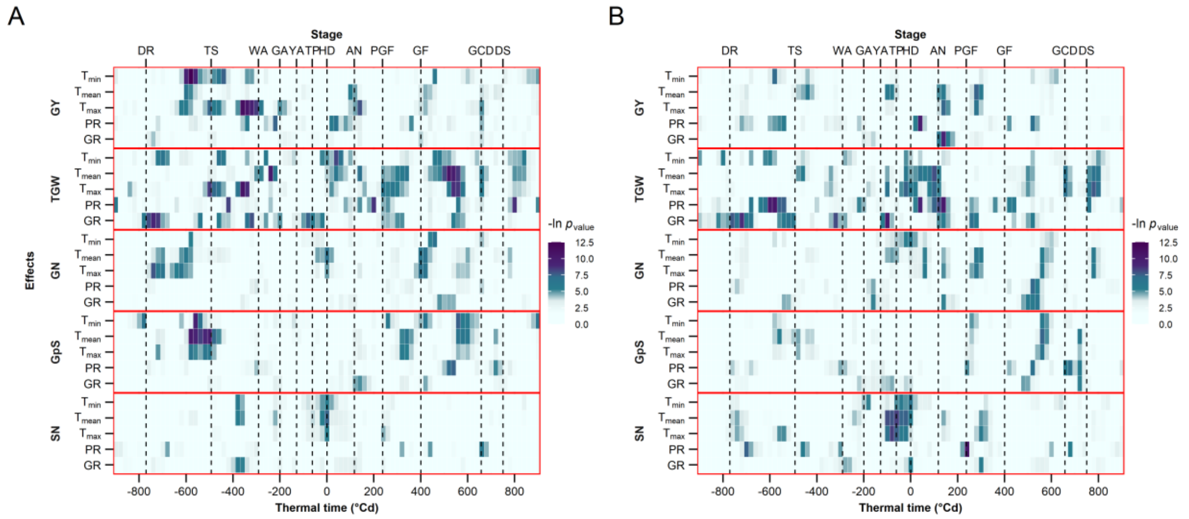

Supplementary Fig. 5. **Comparing (A) significance (−ln(*P*) in Fig. 4) and (B) sensitivity between old and modern cultivars.** The significant results (higher −ln *pvalue*) in this figure indicate the responses of yield and yield components (GpS, SN, GN and TGW) to short-term environmental variables (GR, T_{max}, T_{mean}, T_{min} and PR) before and after heading (thermal time = 0 °Cd) have been changed by breeding. Yield and different yield components are separated by solid red lines. AN, anthesis; DR, double-ridge stage; DS, desiccation; GA, green anther stage; GCD, green canopy duration characterized by 50% canopy senescence; GF, grain filling; HD, heading stage; PGF, pre-grain filling; TS, terminal spikelet stage; WA, white anther stage; YA, yellow anther stage.
