# Supplementary material for: Plasticity of source-sink dynamics contributes to wheat yield stability
Source: Nat Commun. 2026 Apr 24;17:3781. doi: 10.1038/s41467-026-72330-x (PMC13109390; doi:10.1038/s41467-026-72330-x)
Supplement: Supplementary file 3 — Description of Additional Supplementary Files [file 41467_2026_72330_MOESM3_ESM.pdf]

**Description of Additional Supplementary Files**

File Name: Supplementary Data 1
Description: brief summary of cultivar information from different data sources.

File Name: Supplementary Data 2
Description: Summary of traits, their abbreviations, meanings, data sources, phenological stages, range of year releases, number of environments, absolute and relative breeding progresses in this data-synthesis. All traits are grouped in four category: 1) Source traits, 2) Source/Sink traits, 3) Sink traits and 4) Other traits. Different aspects of each category are further classified into sub-categories. Number of studied environments (n) is summed up over data sources. Data of each trait was obtained from different time points throughout different developmental phases in terms of BBCH code (Stage). Absolute breeding progress describe the slope between the trait values and year of release (Supplementary Fig. 2A) relative breeding progress estimate the differences between modern (2010) and old (1970) cultivars in terms of ratio (Supplementary Fig. 2B).

File Name: Supplementary Data 3
Description: Full Ghat selection statistics across environments. For each trait and environment combination, the permutation-based Ghat statistics, associated p-value, and Pearson correlation coefficient (r) between marker effects and allele frequency changes are reported. The Ghat statistic tests for directional selection based on aggregated marker effect - allele frequency covariance. All p-values were obtained from permutation tests. Significant results ($p < 0.1$) correspond to colored cells in Fig. 2. Datasets are distinguished by abbreviations: BRIWECS1 (BW1); BRIWECS2 (BW2); Lichthardt (LH); PhenoPlast (PP); WheatSouSi1 (WS1); WheatSouSi2 (WS2). Growing environments are labelled using combinations of treatments and plant part abbreviations. Abbreviations of treatments in field trials: high nitrogen (HN); low nitrogen (LN); no fungicide (NF); with fungicide (WF); rainfed (RF); irrigated (IR); rainout shelter (RO). Abbreviations of treatment in growth chamber: control conditions (CC); fluctuating light (FL); fluctuating temperature (FT); high temperature (HT); low temperature (LT); high light (HL); low light (LL); Abbreviations of treatment in green house: water stress (WS); well-watered (WW). Abbreviation of plant parts: the third leaf (L3); the sixth leaf (L6); under-side of the leaf (abaxial); upper-side of the leaf (adaxial).
