# Supplementary material for: Plasticity of source-sink dynamics contributes to wheat yield stability
Source: Nat Commun. 2026 Apr 24;17:3781. doi: 10.1038/s41467-026-72330-x (PMC13109390; doi:10.1038/s41467-026-72330-x)
Supplement: Supplementary file 5 — Reporting Summary [file 41467_2026_72330_MOESM5_ESM.pdf]

nature portfolio

Corresponding author(s): Tsu-Wei Chen

Last updated by author(s): Feb 27, 2026

Reporting Summary

Nature Portfolio wishes to improve the reproducibility of the work that we publish. This form provides structure for consistency and transparency in reporting. For further information on Nature Portfolio policies, see our Editorial Policies and the Editorial Policy Checklist.

Statistics

For all statistical analyses, confirm that the following items are present in the figure legend, table legend, main text, or Methods section.

n/a	Confirmed	
☐	☒	The exact sample size (*n*) for each experimental group/condition, given as a discrete number and unit of measurement
☐	☒	A statement on whether measurements were taken from distinct samples or whether the same sample was measured repeatedly
☐	☒	The statistical test(s) used AND whether they are one- or two-sided<br>*Only common tests should be described solely by name; describe more complex techniques in the Methods section.*
☒	☐	A description of all covariates tested
☒	☐	A description of any assumptions or corrections, such as tests of normality and adjustment for multiple comparisons
☐	☒	A full description of the statistical parameters including central tendency (e.g. means) or other basic estimates (e.g. regression coefficient) AND variation (e.g. standard deviation) or associated estimates of uncertainty (e.g. confidence intervals)
☐	☒	For null hypothesis testing, the test statistic (e.g. *F*, *t*, *r*) with confidence intervals, effect sizes, degrees of freedom and *P* value noted<br>*Give P values as exact values whenever suitable.*
☒	☐	For Bayesian analysis, information on the choice of priors and Markov chain Monte Carlo settings
☒	☐	For hierarchical and complex designs, identification of the appropriate level for tests and full reporting of outcomes
☒	☐	Estimates of effect sizes (e.g. Cohen's *d*, Pearson's *r*), indicating how they were calculated

Our web collection on statistics for biologists contains articles on many of the points above.

Software and code

Policy information about availability of computer code

Data collection	No software and code were involved in data collection.
Data analysis	All data analyses were conducted from customized code using R-environments (v.4.4.1). R Package Ghat (v0.2.0) and rrBLUP (v.4.6.3) were used to study the genetic selection signal

For manuscripts utilizing custom algorithms or software that are central to the research but not yet described in published literature, software must be made available to editors and reviewers. We strongly encourage code deposition in a community repository (e.g. GitHub). See the Nature Portfolio guidelines for submitting code & software for further information.

Data

Policy information about availability of data

All manuscripts must include a data availability statement. This statement should provide the following information, where applicable:

- Accession codes, unique identifiers, or web links for publicly available datasets
- A description of any restrictions on data availability
- For clinical datasets or third party data, please ensure that the statement adheres to our policy

The complete sets of BLUEs values for the analyses in this study have been deposited in the repository of Humboldt Universität zu Berlin, the institute of the first and corresponding authors under https://scm.cms.hu-berlin.de/intensive-plant-food-systems-public/wang-et-al-2026-nature-communications

Research involving human participants, their data, or biological material

Policy information about studies with human participants or human data. See also policy information about sex, gender (identity/presentation), and sexual orientation and race, ethnicity and racism.

Reporting on sex and gender	not applicable
Reporting on race, ethnicity, or other socially relevant groupings	not applicable
Population characteristics	not applicable
Recruitment	not applicable
Ethics oversight	not applicable

Note that full information on the approval of the study protocol must also be provided in the manuscript.

Field-specific reporting

Please select the one below that is the best fit for your research. If you are not sure, read the appropriate sections before making your selection.

[x] Life sciences   [ ] Behavioural & social sciences   [ ] Ecological, evolutionary & environmental sciences

For a reference copy of the document with all sections, see nature.com/documents/nr-reporting-summary-flat.pdf

Life sciences study design

All studies must disclose on these points even when the disclosure is negative.

Sample size	There are different data sets involved in our analyses so the sample size is data set dependent. Ranging from 2-30. Field trials were conducted in three seasons (2014–2015, 2015–2016, and 2016–2017, 2017-2018 , 2018-2019 and 2022-2023) at six different locations with 229 wheat cultivars. Field trials were conducted in yield plots (4.5–12 m2) depending on site-specific sowing and harvesting machinery.<br>Experimental design was a randomized block design with two replications and management intensities. Full details are shown in Supplmentary Data S2. Sample size per year, location, treatment and genotype = 2-3, corresponding the maximal capacity of the field experiments.<br>We report the genetic and breeding trends in each environment (year, location and treatment combination). In this study, we conduct the statistics of these trends across environment. This means, the reported values (in Fig. 1-4) are already the median of repeated experimental outcomes. We show confidence interval to very the reproducibility of the experimental findings.
Data exclusions	We carefully check the data quality before starting the analyses. Unrealistic data points were excluded (e.g. thousand kernal weight = 100g, less than 0.1% of the whole dataset)
Replication	For the analyses of breeding progress, number of replication range up to 100 environments, depend on traits (detailed in Fig.1 and Table S2). Some traits that are difficult to measure were only measured in one environment.
Randomization	Randomized block design.
Blinding	Yes. All experimental unit is barcoded as an identical number within an experiment (e.g. S1128), so during the data collection, the investigators do not know the genetic identity of the measured plots or plants.

Reporting for specific materials, systems and methods

We require information from authors about some types of materials, experimental systems and methods used in many studies. Here, indicate whether each material, system or method listed is relevant to your study. If you are not sure if a list item applies to your research, read the appropriate section before selecting a response.

Materials & experimental systems

n/a	Involved in the study	
[x]	[ ]	Antibodies
[x]	[ ]	Eukaryotic cell lines
[x]	[ ]	Palaeontology and archaeology
[x]	[ ]	Animals and other organisms
[x]	[ ]	Clinical data
[x]	[ ]	Dual use research of concern
[ ]	[x]	Plants

Methods

n/a	Involved in the study	
[x]	[ ]	ChIP-seq
[x]	[ ]	Flow cytometry
[x]	[ ]	MRI-based neuroimaging

Dual use research of concern

Policy information about dual use research of concern

Hazards

Could the accidental, deliberate or reckless misuse of agents or technologies generated in the work, or the application of information presented in the manuscript, pose a threat to:

No	Yes	
☒	☐	Public health
☒	☐	National security
☒	☐	Crops and/or livestock
☒	☐	Ecosystems
☒	☐	Any other significant area

Experiments of concern

Does the work involve any of these experiments of concern:

No	Yes	
☒	☐	Demonstrate how to render a vaccine ineffective
☒	☐	Confer resistance to therapeutically useful antibiotics or antiviral agents
☒	☐	Enhance the virulence of a pathogen or render a nonpathogen virulent
☒	☐	Increase transmissibility of a pathogen
☒	☐	Alter the host range of a pathogen
☒	☐	Enable evasion of diagnostic/detection modalities
☒	☐	Enable the weaponization of a biological agent or toxin
☒	☐	Any other potentially harmful combination of experiments and agents

Plants

Seed stocks	All studied cultivars are/were commercial cultivars. Seeds are available upon request.
Novel plant genotypes	not applicable
Authentication	not applicable
